# Demographic and Environmental Factors Associated with Mental Health: A Cross-Sectional Study

**DOI:** 10.3390/ijerph14040431

**Published:** 2017-04-17

**Authors:** Jayeun Kim, Ho Kim

**Affiliations:** 1 Institute of Health and Environment, Seoul National University, Seoul 08826, Korea; kimjayeun@gmail.com; 2 Department of Public Health Science, Graduate School of Public Health, Seoul National University, Seoul 08826, Korea

**Keywords:** community health, cross-sectional study, demographic character, greenness, mental health

## Abstract

Relevant demographic and environmental conditions need to be understood before tailoring policies to improve mental health. Using community health survey data from 25 communities in Seoul, 2013, cross-sectional associations between mental health and community level environments were assessed. Mental health outcomes (self-rated stress levels (SRS) and depressive symptoms (DS)) were analyzed. Community environmental factors included green space, green facilities, and annual PM_10_ level (AnnPM_10_); socio-demographic factors included sex, age, education, labor market participation, comorbidity, sleep hours, physical activity, smoking, and drinking. A total of 23,139 people with the following characteristics participated: men (44.2%); age groups 19−39 (36.0%), 40−59 (39.4%), 60−74 (19.2%), and 75+ (5.4%). Women had higher odds ratios (OR) for SRS [OR 1.22, 95% Confidence interval (CI) 1.17–1.27] and DS [OR 1.55, 95% CI 1.42–1.71]. Regular physical activity predicted SRS [OR 0.90, 95% CI 0.84–0.95] and DS [OR 0.98, 95% CI 0.88–1.10]; current smoking and drinking were adversely associated with both SRS and DS. Higher accessibility to green space (Q4) was inversely associated with DS [OR 0.89, 95% CI 0.81−0.97] compared to lower accessibility (Q1). AnnPM_10_, annual levels for particles of aerodynamic diameter <10 µm (PM_10_), among communities was associated with poorer SRS [OR 1.02, 95% CI 1.00–1.04] by 10 μg/m^3^ increases. Therefore, both demographic and environmental factors should be considered to understand mental health conditions among the general population.

## 1. Introduction

Concerns about mental health have been growing worldwide and mental health conditions are reported to depend on many factors, including not only demographic factors [1,2,3] such as socio-economic factors [4,5] and health behaviors, but also ecological environmental factors [6,7,8,9]. However, previous studies on the relationships between mental health and associated factors have usually focused on either objective demographic factors including socio-economic conditions, environmental factors, subgroup populations among inpatients, or on specific age groups such as younger generations [8] or the elderly [2,6,10].

Already, some studies have reported that perceptions of low social status in adults may be involved in the pathogenesis of depression [4] and an index of socio-economic status seems to predict depression symptomatology across European countries [5]. However, most studies related to mental health have focused on depression, and there are few studies related to stress or subjective mental health outcomes. In addition, the health behavior factors may be associated with mental health outcomes in both directions; for instance, physical activity or sufficient sleep may be beneficial while smoking or drinking is harmful.

Evidence for the relationship between availability or quality of green areas in neighborhood environments and mental health has been established through various studies. In several studies, living conditions with surrounding greenery were suggested to have benefits for improving mental health [9,11,12,13]. In addition, community based research on mental health has reported adverse health effects of increased levels of air pollution [8].

Seoul is a megacity in the Republic of Korea composed of 25 districts, with one-fifth of the total national population living there. Although the general health status of Seoul citizens may greatly depend on demographic factors, regional characteristics cannot be ignored. Regarding regional characteristics, environmental conditions such as levels of air pollution or greenness have been regulated by environmental policy. For instance, the Seoul government has promoted policies for decades to increase the number and quality of green spaces and to improve air quality. Although the Seoul government has been attempting to improve the environmental quality in Seoul, improvements in citizen’s living conditions in terms of common mental health outcomes may not be measurable as many studies focusing on mental health outcomes have relied on medical service utilization with doctors’ clinical diagnoses. In addition, accessibility and level of exposure to environmental factors varies by community region and the effect of greenness on health may differ across individual demographic characteristics as much as environmental factors. Consequently, it is necessary to examine conditions at the community as well as individual levels to improve the mental health of local community residents.

Therefore, in terms of determining population based general mental health status and improving common mental health, it is necessary to explore and understand both highly relevant demographic and environmental factors before introducing tailored policies to improve mental health. Accordingly, we investigated the relationships between individual mental health and demographic factors as well as various environmental factors; we also investigated the moderation effects between some health behavioral factors and environmental factors to assess whether health behaviors were modified by environmental factors using community health survey data. Regarding community health survey data, the Korean government established the Community Health Survey (CHS) in 2008; this survey was designed to investigate health status and its determinants among the adult population in Korea. As the CHS targeted the general population, exploring the association between demographic and ecological environmental factors and common mental health outcomes such as stress level or experience of depression symptoms depends on subjective sentiments.

## 2. Materials and Methods

### 2.1. Study Population

We used the 2013 Community Health Survey (CHS) data in Korea; this survey has been conducted periodically since 2008 by the Korea Center for Disease Control and Prevention (KCDC) and public health centers. The purpose of the Survey is to assess health status, as well as related behavior and determinants, in the Korean population and to produce community-based comparable health statistics at various spatial scales. Annually, approximately 230,000 people participate in the CHS and these participants are randomly sampled using regional stratification every year. As it is based on a cross-sectional study, the participants are newly sampled each year and the target population of the CHS are adults (≥19 years) living in each community. The CHS study has been accompanied by the Community Health Survey Quality Control and Evaluation (CHS-QCE) study throughout the survey process. The objective of CHS-QCE was to establish a validation system for the CHS to ensure the accuracy and reliability of the community health survey [14]. For instance, the CHS-QCE developed a standardization manual for the quality management survey and adopted a Quality Control Index with nine components: replacement of sample household, completion of household, answer rejection, agreement of answer, compliance with survey guidelines, and four detailed indicators related to time compliance in the conduct of the survey. In addition, telephone checks by a third partner were conducted and response concordance was evaluated between the interviewer-assisted survey and the telephone survey by the third party.

In our study, we restricted the study populations to Seoul residents residing in 25 communities, which we call “Gu” as an administrative district; the total sample size is 23,139, and the sample size of each community is approximately 900. Because this survey was conducted on samples selected out of the total population, in the estimation stage, we applied individual weights provided along with survey data from KCDC. This study was approved for exemption by the Institutional Review Board of Seoul National University (IRB No. E1611/001-003).

### 2.2. Demographic Factors and Mental Health

The CHS is conducted by trained interviewers based on a protocol and questionnaires; the data collection mode is a computer-assisted personal interview (CAPI). This CHS data provides individual-level information on health status and its determinants. The questionnaires consist of a personal survey including health behavior, chronic disease, injury, quality of life, immunizations and screening, utilization of health services, socio-demographic characteristics, and a family survey including the type of house and household income. The CHS was solely conducted by personalized interviewers using CAPI and the questionnaires were used. The questionnaires consisted of common and region specific questions; common questionnaires were applied to all participants and only a few questionnaires varied at the level of survey regions. The variables we used for demographic factors included sex, age, education, labor market participation, comorbidity, sleep hours, physical activity, smoking, and drinking; for mental health status, we used the relevant part of the common questionnaires.

The participants’ ages were grouped into 19–39, 40–59, 60–74, and ≥75 years; education levels were grouped into uneducated or elementary school, middle school or high school, university including colleges, and master’s course or above. Labor market participation was defined using a question (Have you worked more than one hour for the last one week for income purposes or ever worked as a family worker?) with two response options (Yes, No). When the participants answered “Yes”, they were considered to participate to in the labor market and participants on temporary leave were counted as labor market participants. We also considered comorbidities including hypertension, diabetes, dyslipidemia, stroke, and arthritis; if participants were diagnosed by a doctor and currently under treatment for at least one disease, they were grouped into any comorbidity and others were grouped into non-comorbidity. Physical activity was defined using a question (In the last week, how many days did you have 10 min or more of intense physical activity, which involved breathing very hard or harder than usual?) with continuous responses between 0 to 7 days. Vigorous (high intensity) physical activity included activities such as running (jogging), climbing, fast biking, swimming fast, soccer, basketball, jumping rope, squash, singles tennis, as well as occupational activities such as carrying heavy objects.

For the response variables of mental health outcomes, we selected self-rated stress levels and experience of depression. Self-rated stress level was defined using a question (How much stress do you usually feel during daily life?) with four response options (very much, a lot, a little bit, and rarely). When participants answered “very much” or “a lot”, they were grouped into higher levels of stress and the remainder were grouped into lower levels of stress. Depression experience was defined using a question (Have you ever felt sadness or despair in the last two consecutive weeks in the recent year?) with two response options (Yes, No). When participants answered “Yes”, they were grouped into the depression experienced group and the remainder were grouped into the depression not experienced group.

### 2.3. Environmental Variables

We used air pollutant data and greenery data as representations of community based environmental variables. First, air pollutant data was collected from the National Institute of Environmental Research and we used the PM_10_ (µg/m^3^) levels as the units of annual average concentrations in the analysis. Using the hourly measured PM_10_ levels in each community site, we calculated daily mean levels of PM_10_ and averaged out daily values into annual levels for each community between 2005 and 2013. In addition, we applied simple linear regression with the observed nine annual levels of PM_10_ from 2005 to 2013 and estimated the trend of the annual level of PM_10_ according to 1-year increases in each community.

We obtained park and green area data for the study year 2013 from the environmental section with statistical data provided by the Seoul Metropolitan government. Seoul statistics provided information about the total space of the greenness area, total number of green facilities, and general park area, city park area, and residence area park per capita. For the reliability of the greenness data, we compared the annual values of the variables we used in the analysis since 2004 and observed the number of green facilities and greenness area that have steadily increased. According to the definition of the data from Seoul Statistics, a greenness area is a comprehensive area including green facilities areas, general green areas, and riverside green areas. A green facilities area is a green area designated by the city management plan, a general green area includes green areas around the roads and living areas, and a riverside green area includes green areas of riversides such as the Jungrang Stream and Yangjae Stream. We used three variables for park per capita; a general park area includes city park areas and residential area parks. A city park area is the area as stipulated in the Urban Parks and Greenery Act and residential area parks include parks that are easy to access and often used in the neighborhood. Thus, some areas for city park areas and residential area parks overlap.

### 2.4. Statistical Analysis

The CHS data were collected by surveys of the sampled populations upon regional stratification and the data were supplied with individual weights for each subject. Initially, we investigated the relationships between the health outcomes using ordinary logistic regression and then applied survey based logistic regression analysis to assess the population-based associations between mental health, specifically self-rated stress levels and experience of depression, and individual demographic and socio economic conditions, and community based environments. As long as the outcome of mental health was thought to be related to demographic characteristics including conditions of comorbidities, health behaviors, and socio economic status (SES), we specified the initial model to include demographic characteristics and SES as covariates and observed the association with mental health outcomes. Afterwards, we conducted further multivariate analysis by fitting each of the environmental factors into the initial model and estimated the effect of these environmental factors on mental health outcomes. The environmental related factors were used for greenness; these included greenness area (km^2^), general park area per capita (m^2^), city park area per capita (m^2^), residence area park per capita (m^2^), number of green facilities and air pollutants, annual levels of PM_10_ in the year 2013, and the estimated trend of the annual level of PM_10_ between 2005 and 2013. In addition, we investigated the moderation effect using interaction terms between specific health behavioral factors and one of the environmental factors in ordinary logistic regression models that fit all the demographic factors.

There were missing values for some variables including education, labor market participation, smoking, and drinking; we excluded missing observations during the regression analysis. The number of missing observations was few compared to the total study sample and the most frequent missing variable was education, with 50 participants (0.22%), while other variables had missing values for one or three participants each.

All results are presented as odds ratios (OR) with 95% confidence intervals (CIs). All procedures were conducted using SAS version 9.4 (SAS Institute, Inc., Cary, NC, USA) and all figures were modeled using R 3.1.1 (Open Source) (The Comprehensive R Archive Network: http://cran.r-project.org). All statistical tests were 2-sided and a *p*-value < 0.05 was considered statistically significant.

## 3. Results

### 3.1. Demographic Characteristics and Descriptive Statistics

There were 23,139 participants in the study [men 10,231 (44.2%), women 12,908 (55.8%)] and age groups consisted of 19−39 [8340 (36.0%)], 40–59 [9117 (39.4%)], 60–74 [4438 (19.2%)], and 75+ [1253 (5.4%)] (Table 1). The average sleep hours per day during a recent week were classified into three groups: <6 [4236 (18.3%)], 6–7 [7668 (33.1%)], and ≥7 [11,235 (48.6%)]. Prevalence of hypertension was 18.6% in men (1907), and 17.0% in women (2194). The majority of participants did not engage in physical activity [16,617 (71.8%)] but more than half of the study sample were active in the labor market: total 61.2% (14,168), men 75.0% (7669), and women 50.3% (6499). Regarding mental health problems, 28.1% (6513) reported higher self-rated stress levels and 7.2% (1675) reported depression experiences. Statistically significant gender differences were observed for all demographic and SES variables (*p* < 0.05); men tended to practice intensive physical activity more frequently (χ^2^ = 734.8, *p* < 0.0001) and participated in the labor market more actively (χ^2^ = 1458.2, *p* < 0.0001) than women.

**Figure 1:**
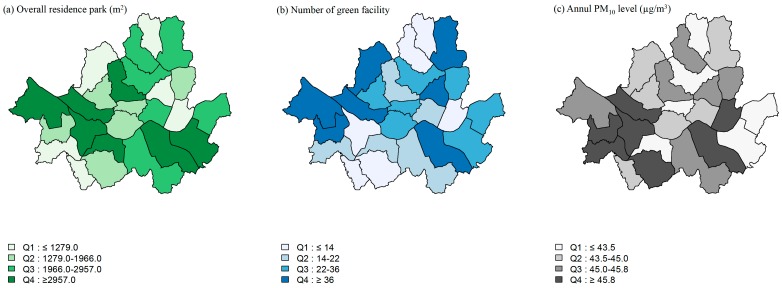
Quartile distribution of residence park, number of green facilities, and annual PM_10_ levels among 25 communities in Seoul, Korea, 2013.

Certain environmental factors are described in Figure 1 using quartile division and descriptive statistics; correlations for the greenery related variables and particulate matter (PM_10_) related variables are presented in Table 2. The higher level of overall residential park areas tended to be centralized while the number of green facilities tended to form a boundary in Seoul; the levels of annual PM_10_ were higher in the southern part of Seoul (Figure 1).

The mean annual level of PM_10_ among the 25 communities was 44.6 µg/m^3^ (standard deviation; SD 1.6 µg/m^3^). Slopes for the annual level of PM_10_ from 2005 to 2013 mostly decreased among the 25 communities; the mean of the beta slope was −0.3 (SD = 0.08) and it was positively correlated with the annual levels of PM_10_, ρ = 0.13 (*p*-value > 0.05). Greenery related variables had negative but non-significant correlations with annual PM_10_ related variables. Furthermore, city park area per capita (ρ = 0.75, *p*-value < 0.001) and living area per capita (ρ = 0.53, *p*-value < 0.05) had significant positive correlations with the general park area per capita. The varied greenness area by community level had positive but non-significant correlations with general parks per capita (ρ = 0.16, *p*-value > 0.05), city park areas per capita (ρ = 0.30, *p*-value > 0.05), and residential area parks per capita (ρ = 0.19, *p*-value > 0.05). However, the greenness area at the community levels had a negative but non-significant correlation with the number of greenery facilities by community level (ρ = −0.19, *p*-value > 0.05).

### 3.2. Associations between Mental Health and Demographic and Environmental Factors

The associations for demographic characteristics were explored in multivariate models and the results are presented in Table 3. Women had considerably poorer mental health outcomes for both self-rated stress levels [OR 1.22, 95% CI 1.17–1.27] and depression experience [OR 1.55, 95% CI 1.42–1.71] compared to men.

Regular physical activity at least 3 days per week was associated with decreased self-rated stress levels [OR 0.90, 95% CI 0.84–0.95] and depression experience [OR 0.98, 95% CI 0.88–1.10]. Inactive participation in the labor market was associated with lower self-rated stress levels [OR 0.87, 95% CI 0.83–0.90] but with higher rates of depression experience [OR 1.22, 95% CI 1.14–1.30].

We observed increased associations with education levels that were lower than masters’ degree among subjects with depressive symptom experiences while inconsistent results were detected among subjects with higher self-rated stress levels. Participants with at least one comorbidity from hypertension, diabetes, dyslipidemia, stroke, and arthritis were more likely to have poorer mental health outcomes, that is, higher self-rated stress levels [OR 1.10, 95% CI 1.05–1.16], and more depression experience [OR 1.07, 95% CI 0.99–1.15]. Current smoking and drinking tended to be associated with poorer mental health outcomes.

In the multivariate models fitted with the demographic characteristics shown in Table 3, we fitted, in steps, one of the community based environmental factors including greenness areas, general park areas, city park areas, residential area parks, number of green facilities, annual PM_10_ levels, and absolute values of estimated PM_10_ slopes between 2005 and 2013 (Table 4). The greenness related variables were divided by quartiles and grouped into three, with quartile 1, quartiles 2–3, and quartile 4. None of them demonstrated any relationship with mental health outcomes except for the number of green facilities. Communities with the most green facilities (Q4) had a decreased association with depression experienced [OR 0.89, 95% CI 0.81–0.97] compared to communities with the least (Q1). Furthermore, annual levels of PM_10_ in each community were adversely associated with subjective stress levels [OR 1.02, 95% CI 1.00–1.04] by increases of 10 μg/m^3^; PM_10_ β was non-significantly associated with decreased stress levels [OR 0.78, 95% CI 0.52–1.18] and depression experience [OR 0.79, 95% CI 0.40–1.57] by increases of 0.1 units.

### 3.3. Moderation Effects between Health Behavior and Environmental Factors

**Figure 2:**
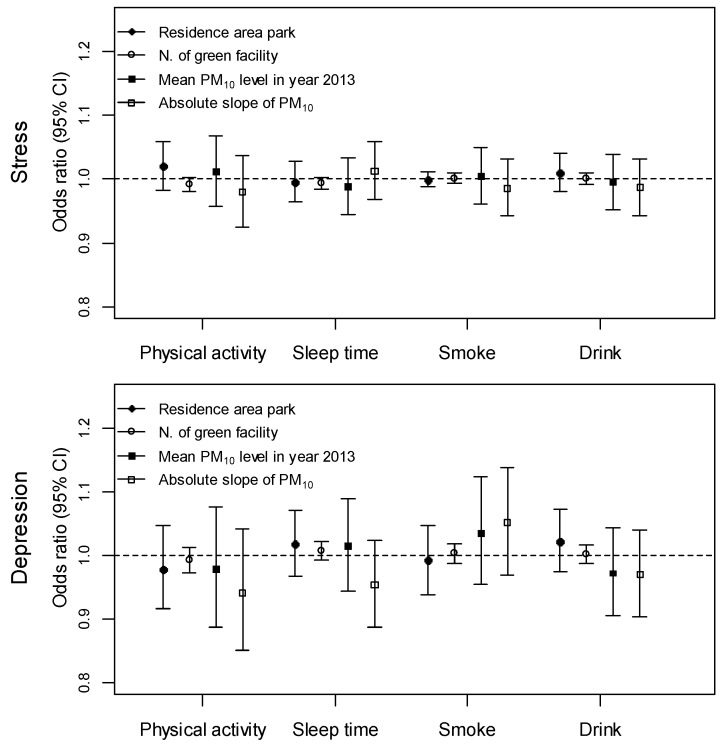
Effect modification between individual health behavior and community based environments are presented. The modification was estimated using interaction terms and the reference groups of physical activity, sleep time, smoke, and drink are less than 2 days of physical activity during a week, less than 6 h of sleep in a day, currently not smoking, and currently not drinking, respectively. Odds ratio (OR) with 95% confidence interval (CI) was estimated for each environment with certain unit increases; residential area park (3 m^2^), number of green facilities (5 units), annual mean level of PM_10_ (2 µg/m^3^), and absolute slope of PM_10_ (0.1 unit).

We investigated the moderation effects between some health behaviors and environmental factors using interaction terms in the ordinary logistic regression model adjusted for demographic characteristics as shown in Table 1; the results are presented in Figure 2. The chosen health behaviors were physical activity, sleep hours, smoking, and drinking. These were modified into dichotomous variables: physical activity days per week (3− vs. 3+), sleep hours per day (6− vs. 6+), smoking (current vs. never or previous), and drinking (current vs. never or previous). Among the environmental variables, residential area parks, number of green facilities, annual levels of PM_10_, and absolute estimates of PM_10_ levels were reviewed and none of them were significantly moderated by health behavior. However, increased associations were detected between depression experience and annual PM_10_ levels [OR 1.04, 95% CI 0.95–1.12] or PM_10_ slope [OR 1.05, 95% CI 0.97–1.14] among current smokers by unit increases of 2 μg/m^3^ and 0.1 μg/m^3^ per year, respectively.

## 4. Discussion

### 4.1. Principal Findings

We investigated the relationships between various demographic and environmental factors, and individual mental health using community health survey data. We observed that women had considerably poorer mental health outcomes for both self-rated stress levels and depression experience, and inactive participation in the labor market was associated with lower self-rated stress levels but with higher rates of depression experience. We observed contradictory associations with education levels between depressive symptom experiences and self-rated stress levels and health behaviors; smoking and drinking were also associated with mental health outcomes while regular physical activity more than 3 days a week had a beneficial effect on the mental health outcomes.

We regarded the number of green facilities in each community as a marker of accessibility to green spaces because higher levels of green facilities may allow for the residents’ exposure to higher levels of greenness. Accessibility of green spaces was associated with improvements in mental health, while levels of particulate matter (PM_10_) were associated with poorer mental health outcomes. In addition, we investigated the moderating effects between some of the health behaviors and environmental factors. Through applying interaction terms, we observed whether the associations between mental health outcomes and environmental factors were moderated by individuals’ health behavior such as practicing a regular physical activity, and overall sleep time. However, a beneficial effect on mental health of green and natural spaces was not detected in the moderation analysis.

### 4.2. Associations between Mental Health and Demographic Characteristics

The associations between demographic characteristics and mental health outcomes were of main interest in this study and we observed varied outcomes compared with other studies [1,2,3,15,16]. We found that women were more likely to experience higher levels of stress and depressive symptoms than men [1,2]. Furthermore, we observed increased associations with education levels lower than masters’ degrees among subjects with depressive symptom experiences, as we utilized education level and labor market participation as markers of socio-economic status [1,2,3]. However, levels of stress varied by education, as subjects with intermediate levels of education were less likely to experience higher levels of stress. A predictable but interesting result was that labor market participation had contrasting effects depending on the type of mental health outcome considered. Subjects participating in the labor market experienced more stress but were less likely to experience depressive symptoms [3]. This result suggests that encouraging labor market participation can reduce depression prevalence while at the same time labor-level adjustment is required to reduce the intensity of stress from labor market participation.

Adverse health effects were observed for smoking [17,18,19] and drinking [20,21,22] and current smokers were more likely to experience higher levels of stress and depressive symptoms. As this study used a cross-sectional design, the results should not be interpreted in causal terms, such as whether adverse health behavior resulted in poorer health and unstable mental status or vice versa. However, as strong associations were detected between those health behaviors and mental health, it should be noted that people with current smoking and drinking behaviors are less likely to cope well with their mental difficulties [17].

In addition to adverse mental health effects associated with demographic characteristics, beneficial factors for mental health were also observed. Longer sleeping times improved mental health and more than 7 h of sleep per day lowered the likelihood of experiencing stress or depressive symptoms. In addition, regularly practicing physical activity more than 3 days a week was associated with lower stress levels and a similar relationship was detected with depressive symptom experiences. Inverse relationships between sleep hours [23,24,25] or physical activity and adverse mental health outcomes have been suggested in several studies. Hashizume et al. [24] stressed the importance of sufficient sleep time because insufficient sleep can cause impaired cognitive function and mental disorders such as depression; Lee et al. [23] reported gender-specific patterns such as significant associations in women with stress and depressive symptoms, and in men with stress. The study examined the effects of sleep deprivation on serum cortisol levels and mental health among military servicemen in China and suggested that sleep deprivation could significantly increase serum cortisol level and may affect mental health among servicemen [25]. Overall, the outcomes of the present study defined susceptible populations based on individual demographic factors such as biological information, health behaviors, and socio-economic status, and provided supportive evidence of the importance of demographic characteristics on mental health.

### 4.3. Associations between Mental Health and Greenness

For the environmental factors, we observed a marginal but protective relationship on mental health by green facilities. Although the outcome may be biased due to limitations of the study design, such as being a cross-sectional study, several longitudinal cohort studies have reported similar results. For instance, James et al. [26] examined the prospective association between residential greenness (Normalized Difference Vegetation Index (NDVI)) and mortality using data from the US-based Nurses’ Health Study prospective cohort. This study reported that higher levels of green vegetation were associated with decreased mortality and observed a stronger association between greenness and mortality among participants with higher levels of physical activity. However, our study was not able to apply the NDVI for greenness because of limited data sources. Instead, we utilized information about parks and the accessibility of green facilities. Some similar studies have already been conducted using parks in urban areas. For instance, a beneficial effect from the exposure to green space on disease risk and mortality was suggested, although the association varied according to the combination of area income deprivation and urbanity [27]. Additionally, Garter et al. [28] reported that access to parks and green spaces within residential neighborhoods has been shown to be an important pathway to generating better physical and mental health for individuals and communities using mixed methods in Perth, Western Australia.

In this study, we regarded the number of green facilities as a marker of accessibility to green spaces, which is defined as greenness or green space. Although some communities have larger green spaces than others, the green spaces mostly consist of moderate mountains. These specific conditions of greenness may limit accessibility to green environments for populations who do not like hiking or climbing mountains or who having physical or ambulatory difficulties. Nonetheless, we found evidence for a protective association between access to greenness and levels of stress and this result supports the consistent evidence that greenness exposure is protective against adverse mental health outcomes by a cross-sectional design. Furthermore, other cross-sectional evidence in the UK found that living closer to urban green spaces, such as parks, was associated with lower mental distress [29]. In summary, although our data limited our ability to fully investigate the relationship between mental health and greenness, similar outcomes have been found for green facilities closely related with greenness in urban communities.

### 4.4. Associations between Mental Health and Particulate Matter Levels

We observed a marginal but adverse relationship on mental health of the annual level of particulate matter (PM_10_) as another ecological environmental variable. Although, the mechanism whereby particulate matter (PM_10_) causes health effects has not been fully elucidated, much epidemiological evidence supports the adverse association of cardiopulmonary morbidity and mortality with exposure to airborne particulate matter [30,31,32,33] and this adverse evidence has been extended to mental health [7,34,35,36]. In mental health studies related to particulate matter, fine particulate matter (PM_2.5_) was reported as one of the major risk factors linked to poor cognitive function in older, community-dwelling adults in the US [6]. Concerning the impact of PM_2.5_ on mental health, an increase in PM_2.5_ was positively associated with depressive symptoms, and this was significant for the 30-day moving average (OR 1.16; 95% 1.05, 1.29) upon SES adjustment [37].

A study of the short-term effects of PM_10_ levels was also conducted using emergency department visit data in Seoul, Korea; depressive episodes among subjects with either underlying cardiovascular disease, diabetes mellitus, asthma, or depressive disorder were examined and adverse health effects for PM_10_ (OR 1.120; 95% 1.067–1.176) per one standard deviation increase at a lag of 0–3 were observed [38].

We analyzed levels of stress as another variable for mental health status but studies on the association between levels of stress and air pollution are limited. Nonetheless, a longitudinal analysis was conducted for 987 older men participating in the Veterans Administration Normative Aging Study between 1995 and 2007; information about quantified stress experienced in the previous week was collected from the participants [39]. Higher perceived stress was associated with increased risk against PM_2.5_ at moving averages of 1, 2, and 4-week. In this regard, inconsistent associations between air pollution and depression were also presented in four European general population cohort studies. The studies investigated the association between air pollution and depressed mood using residential exposure to particles (PM_2.5_, PM_2.5_ absorbance, PM_10_) and nitrogen dioxide (NO_2_), which was estimated using land use regression (LUR) models; heterogeneous results for cohort specific associations between the air pollutants and depressed mood were reported [40]. However, our study may be limited in examining the association between greenness and mental health outcomes due to limitations of the data and the study design. Nonetheless, it adds additional evidence that increasing accessibility to greenness and decreasing the level of PM10 may improve mental health in an urban city.

### 4.5. Study Limitations

Many environmental epidemiology studies regarding greenness and green space access have been conducted, mainly using a vegetation index (typically the NDVI) or outcomes through land use regressions linked to the participants’ addresses. In our study, we obtained community level information using an administrative district level “Gu” but not participants’ addresses. Therefore, it was hard to define greenness exposure on an individual basis. However, we achieved and utilized an alternative that was stable and comparable between communities, specifically, official administrative data collected by the government of Seoul in a database and supplied to the public annually.

Vegetation may buffer exposure to air pollution to reduce the level of PM_10_ but the quality of green spaces was not explored in this study. Vegetation may vary annually and seasonally but we fitted absolute greenness space regardless of seasonal variations of vegetation in the analysis. However, the CHS has usually been conducted over the 3 months between August and October and mental health outcomes might not be affected by seasons because the greenness quality rarely varied during these periods.

As long as we assigned the exposure of PM_10_ based on each participant’s residential area, the participants’ pattern of life was not considered. In cases wherein participants stayed much longer hours somewhere else instead of in their residential area, this factor may have led to misclassification of some participants because of the variability of exposure levels and durations. In addition, we used only one question to assess depressive symptoms with two response options (Yes, No) among the response variables of mental health outcomes. The depressive symptoms were not dependent on a doctor’s diagnosis but based on subjective experiences in the last two consecutive weeks. However, using only one question and a binary response option may have elicited more biased response outcomes than multiple assessment questions or response options.

## 5. Conclusions

This study strengthened the importance of demographic characteristics such as socio-economic status and health behaviors for maintaining a healthier mentality. Although our study only detected a weak relationship between mental health outcomes and environmental factors, increasing accessibility to greenness and decreasing levels of particulate matter on a community basis are beneficial and are important components in improving mental health conditions among urban citizens. Therefore, to improve the mental health of urban citizens, both policy approaches—giving more care to sensitive or vulnerable groups and making the environment better for citizens overall —need to be taken simultaneously because improved environments may bring more opportunities for physical activity, increase social engagement, and improve mental health.

## Figures and Tables

**Table 1:** Descriptive characteristics of survey populations in the Community Health Survey data in Seoul, Korea, 2013.

Variable	Category	Total		Gender				
				Men		Women		χ^2^ (*p*-Value)
		N	%	n	%	n	%	
Total		23,139	100.0	10,231	44.2	12,908	55.8	
Age group	19–39	8321	36.0	3783	37.0	4538	35.2	12.8 (0.0052)
	40–59	9127	39.4	3964	38.7	5163	40.0	
	60–74	4438	19.2	1970	19.3	2468	19.1	
	75+	1253	5.4	514	5.0	739	5.7	
Sleep hours (day/week)	6–	4236	18.3	1771	17.3	2465	19.1	1232.5 (<0.0001)
	6–7	7668	33.1	3615	35.3	4053	31.4	
	7+	11,235	48.6	4845	47.4	6390	49.5	
Comorbidity prevalence	Hypertension	4101	17.7	1907	18.6	2194	17.0	10.6 (0.0012)
	Diabetes	1555	6.7	803	7.8	752	5.8	37.3 (<0.0001)
	Dyslipidemia	1870	8.1	740	7.2	1130	8.8	17.8 (<0.0001)
	Stroke	211	0.9	117	1.1	94	0.7	10.9 (0.001)
	Arthritis	988	4.3	154	1.5	834	6.5	342.9 (<0.0001)
Physical activity (day/week)	0	16,617	71.8	6434	62.9	10,183	78.9	734.8 (<0.0001)
	1–2	3178	13.7	1922	18.8	1256	9.7	
	3+	3342	14.4	1874	18.3	1468	11.4	
Labor market participation	Active	14,168	61.2	7669	75.0	6499	50.3	1458.2 (<0.0001)
	Inactive	8970	38.8	2561	25.0	6409	49.7	
Education	Master’s course	1615	7.0	933	9.1	682	5.3	578.7 (<0.0001)
	University	10,452	45.2	5048	49.3	5404	41.9	
	Middle school or high school	8355	36.1	3548	34.7	4807	37.2	
	Uneducated or elementary school	2667	11.5	681	6.7	1986	15.4	
Smoking	Never	15,064	65.1	3008	29.4	12,056	93.4	10,290.6 (<0.0001)
	Former	3597	15.5	3198	31.3	399	3.1	
	Current	4475	19.3	4023	39.3	452	3.5	
Drinking	Never	2940	12.7	561	5.5	2379	18.4	1141.1 (<0.0001)
	Former	2845	12.3	953	9.3	1892	14.7	
	Current	17,353	75.0	8717	85.2	8636	66.9	
Self-rated stress level	Very much or a lot	6513	28.1	2808	27.4	3705	28.7	4.5 (0.0345)
	Slightly or rarely	16,623	71.8	7422	72.5	9201	71.3	
Depression experience		1675	7.2	517	5.1	1158	9.0	130.6 (<0.0001)

**Table 2:** Community based environmental variables and their correlations in 25 communities, Seoul, Korea, 2013.

Variable	Mean	Greenness Area	General Park Area	City Park Area	Residence Area Park	No. of Green Facilities	Annual
	(STD)	(km^2^)	Per Capita (m^2^)	Per Capita (m^2^)	Per Capita (m^2^)		PM_10_^1^_(_μg/m^3^_)_
Greenness area (km^2^)	501.2 (349.0)						
General park area per capita (m^2^)	17.1 (14.7)	0.16					
City park area per capita (m^2^)	10.9 (9.7)	0.3	0.75 **				
Residence area park per capita (m^2^)	5.7 (3.6)	0.19	0.53 *	0.41 *			
No. of green facilities	29.0 (21.6)	−0.19	−0.14	−0.03	0.06		
Annual PM_10_^1^ (μg/m^3^)	44.6 (1.6)	0.33	−0.19	−0.26	0.08	−0.26	
Estimated PM_10_ slope ^2^	−0.3 (0.08)	−0.05	−0.31	−0.22	−0.17	0.06	0.13

STD, standard deviation; ** *p*-value < 0.001; * *p*-value < 0.05; ^1^ Seoul has 27 air pollution monitoring sites and at least one air pollution monitoring site is allocated in each community called “Gu” as an administrative district; ^2^ Community based PM_10_ slopes were estimated by linear regressions using annual levels of PM_10_ between 2005 and 2013.

**Table 3:** Associations between mental health outcomes and demographic characteristics.

Variable	Category	Odds Ratio (95% Confidence Interval)	
		Self-Rated Stress Level	Depressive Symptom Experience
Sex	Men	1.00	1.00
	Women	1.22 (1.17–1.27)	1.55 (1.42–1.71)
Age	19–39	1.00	1.00
	40–59	1.18 (1.10–1.26)	1.05 (0.94–1.17)
	60–74	0.78 (0.72–0.84)	1.06 (0.94–1.20)
	75+	0.67 (0.59–0.77)	0.82 (0.68–0.98)
Education	Master’s course	1.00	1.00
	Uneducated or elementary school	1.25 (1.13–1.38)	1.30 (1.12–1.51)
	Middle school or high school	0.91 (0.86–0.97)	1.14 (1.03–1.27)
	University	0.92 (0.87–0.98)	0.98 (0.88–1.10)
Labor market participation	Active	1.00	1.00
	Inactive	0.87 (0.83–0.90)	1.22 (1.14–1.30)
Comorbidity	Non comorbidity	1.00	1.00
	Any comorbidity	1.10 (1.05–1.16)	1.07 (0.99–1.15)
Sleep hours (hours/day)	<6	1.00	1.00
	6–7	0.90 (0.86–0.94)	0.85 (0.79–0.92)
	7+	0.72 (0.69–0.75)	0.84 (0.78–0.90)
Physical activity (day/week)	0	1.00	1.00
	1–2	1.03 (0.96–1.10)	1.04 (0.93–1.17)
	3+	0.90 (0.84–0.95)	0.98 (0.88–1.10)
Smoking	Never	1.00	1.00
	Previous	0.98 (0.92–1.05)	1.12 (1.00–1.26)
	Current	1.34 (1.26–1.42)	1.30 (1.16–1.45)
Drinking	Never	1.00	1.00
	Previous	0.95 (0.88–1.02)	1.06 (0.96–1.18)
	Current	1.09 (1.03–1.15)	1.01 (0.92–1.11)

**Table 4:** Associations between self-rated mental health and community based environments.

Variable	Category		Odds Ratio (95% Confidence Interval)	
			Self-Rated Stress Level	Depressive Symptom Experience
Greenness area (km^2^)	Q1	<235	1.00	1.00
	Q2–Q3	235–667	0.90 (0.84–0.97)	0.90 (0.84–0.97)
	Q4	667+	1.08 (0.99–1.17)	1.08 (0.99–1.17)
General park area per capita (m^2^)	Q1	<7.33	1.00	1.00
	Q2–Q3	7.33–22.67	1.00 (0.95–1.04)	1.11 (1.03–1.19)
	Q4	22.67+	1.01 (0.96–1.06)	0.90 (0.82–0.98)
City park area per capita (m^2^)	Q1	<5	1.00	1.00
	Q2–Q3	5–12.31	1.03 (0.98–1.07)	1.01 (0.94–1.09)
	Q4	12.31+	1.00 (0.95–1.05)	0.96 (0.88–1.05)
Residence area park per capita (m^2^)	Q1	3.51−	1.00	1.00
	Q2–Q3	3.51–6.54	0.97 (0.93–1.02)	0.97 (0.90–1.05)
	Q4	6.54+	0.98 (0.93–1.03)	1.04 (0.96–1.13)
No. of green facilities	Q1	<14	1.00	1.00
	Q2–Q3	14–36	0.98 (0.94–1.03)	1.05 (0.98–1.13)
	Q4	36+	1.01 (0.96–1.06)	0.89 (0.81–0.97)
Annual PM_10_^1^			1.02 (1.00–1.04)	1.01 (0.98–1.05)
Absolute estimated of PM_10_ slope ^2^			0.78 (0.52–1.18)	0.79 (0.40–1.57)

^1^ Odds ratio by 10 and 0.1 unit increases respectively for PM_10_ (μg/m^3^), and absolute estimated PM_10_ slope; ^2^ Beta was estimated on the community based environment using the annual level of PM_10_ between 2005 and 2013 and the beta (slope) was calculated by simple linear regression model.
